# Occupational grief and self-compassion among intensive care unit nurses in tertiary hospitals: a cross-sectional study

**DOI:** 10.3389/fpsyg.2026.1862247

**Published:** 2026-09-14

**Authors:** Yuxiao He, Lixin Kang, Jiarui Hou, Xingru Chen, Shuping Gong, Xiaomin Zhao

**Affiliations:** 1 School of Nursing, Shandong Medical and Pharmaceutical University, Yantai, China; 2 Qilu Hospital of Shandong University Dezhou Hospital, Dezhou, China; 3 School of Health, Binzhou Vocational and Technical University, Binzhou, China

**Keywords:** cross-sectional study, intensive care unit nurses, occupational grief, self-compassion, tertiary hospitals

## Abstract

**Background:**

Intensive care unit (ICU) nurses frequently encounter patient suffering and death and may develop occupational grief. Evidence on occupational grief and its association with self-compassion among ICU nurses in China remains limited.

**Objective:**

To describe occupational grief among ICU nurses in tertiary hospitals and examine its relationship with self-compassion.

**Methods:**

In this cross-sectional study, 355 ICU nurses from nine tertiary hospitals in Shandong, China, were recruited by cluster sampling between January and February 2024. Participants completed a general information questionnaire, the Grief State Scale for Nurses, and the Self-Compassion Scale-Short Form. Descriptive statistics, Pearson and Spearman correlation analyses, reliability testing, confirmatory factor analysis, dimension-level non-parametric comparisons, and hierarchical linear regression were performed.

**Results:**

The mean occupational grief score was 35.22 ± 11.19, and the item mean score was 2.07 ± 0.66. The mean self-compassion score was 37.30 ± 4.03, and the item mean score was 3.11 ± 0.34. Occupational grief was negatively correlated with self-compassion (*Pearson r* = −0.323, *p* < 0.01; *Spearman* ρ = −0.305, *p* < 0.001). In hierarchical regression additionally including education level as a theoretically relevant covariate, poorer self-rated health, self-rated type C personality, self-rated type D personality, and lower self-compassion were independently associated with higher occupational grief. For working-hour categories, working fewer than 8 h per duty day was associated with lower occupational grief compared with working 8–10 h, whereas the >10-h category was not statistically significant in the adjusted model. Self-compassion explained an additional 4.1% of the variance, and the full model explained 20.2% (adjusted *R*^2^ = 0.179).

**Conclusion:**

Occupational grief among ICU nurses was relatively mild based on item mean scores, and Self-compassion was independently associated with occupational grief. Measurement and personality-type findings should be interpreted cautiously, and causal effects require confirmation in longitudinal and intervention studies.

## Introduction

Occupational grief refers to grief responses arising from losses experienced in the workplace (Papadatou, 2000). For nurses, such grief is commonly triggered by the death of patients under their care (Rodriguez et al., 2020). ICU nurses are repeatedly exposed to severe illness, end-of-life care, and patient death, and this exposure may contribute to stress, burnout, and emotional or physical symptoms (Adwan, 2014; Efstathiou and Walker, 2014). Because nurses' grief is often not publicly acknowledged or formally supported, it may become disenfranchised and remain insufficiently addressed (Soklaridis et al., 2019). Occupational grief is conceptually distinct from burnout, compassion fatigue, secondary traumatic stress, and moral distress. Burnout primarily reflects chronic occupational exhaustion and disengagement; compassion fatigue and secondary traumatic stress describe distress related to repeated exposure to others' suffering or trauma; and moral distress arises when clinicians are unable to act in accordance with their ethical judgment. By contrast, occupational grief is centered on emotional responses to workplace losses, particularly patient death. Recent reviews and post-COVID studies have emphasized professional grief and bereavement-support needs among healthcare workers in high-mortality and critical-care settings (Vázquez-Sánchez et al., 2023; Kim et al., 2023; Butler et al., 2024; Phillips et al., 2025; Yin et al., 2025).

In China, empirical studies on nurses' occupational grief remain limited and are mainly descriptive or qualitative (Wang et al., 2023, 2024; Zuo et al., 2022). The novelty of the present study lies in providing multicenter quantitative evidence from ICU nurses in tertiary hospitals in China and in examining self-compassion as a psychological correlate of occupational grief in this population, an association that has not been sufficiently investigated in previous Chinese ICU nursing research. Self-compassion, defined as responding to one's own suffering with kindness, common humanity, and mindful awareness rather than harsh self-judgment, may be relevant for ICU nurses coping with repeated patient loss (Neff, 2023). Theoretically, self-compassion may be associated with lower occupational grief because it is characterized by reduced self-judgment and over-identification and by a more balanced awareness of painful experiences. Prior evidence indicates that self-compassion is associated with lower depression, anxiety, and stress and better psychological wellbeing (Marsh et al., 2018; Zessin et al., 2015; Ford et al., 2017). Personality-related differences may also shape how nurses appraise repeated patient loss and use self-compassionate coping; however, because the present study used only a single self-rated A/B/C/D category, personality was included as an exploratory contextual covariate rather than a validated personality construct. This study aimed to describe occupational grief among ICU nurses in tertiary hospitals and examine its association with self-compassion. We hypothesized that higher self-compassion would be associated with lower occupational grief.

## Materials and methods

### Study design and participants

This cross-sectional study was conducted between January and February 2024. Using cluster sampling, ICU nurses from nine tertiary hospitals in Shandong Province, China, were recruited. The hospitals were not randomly selected; they were tertiary hospitals with eligible ICU units that agreed to participate and were located in different areas of the province. Therefore, hospital selection was partly based on institutional willingness and feasibility, which should be considered when interpreting external validity. After institutional permission was obtained, all eligible ICU nurses in the participating units were invited through the nursing departments. Eligible participants were registered nurses currently employed in ICU units who provided informed consent. Nurses in standardized training or advanced training and nurses who were not on duty because of leave or off-site training were excluded.

Sample size estimation was based on the occupational grief score as the primary outcome. Using the formula for estimating a population mean and assuming α = 0.05, σ = 7.64, and δ = 0.8 on the basis of previous literature (Xu et al., 2024), the minimum required sample size was 350. Allowing for a possible 10% invalid questionnaire rate, the target number of questionnaires to be collected was 385. This target number was used to ensure an adequate final analytic sample and did not indicate that 385 participants were included in the final analysis. The final valid sample was 355 ICU nurses. The study was approved by the Ethics Committee of Binzhou Medical University (IRB: 2024-L002).

### Measures

#### General information questionnaire

The self-designed questionnaire included sex, age, marital status, working hours, professional title, education level, death education training, mindfulness or meditation activities, self-rated health status, self-rated personality type, and other demographic and occupational characteristics. Working hours were collected as self-reported usual total hours per duty day (< 8 h, 8–10 h, or >10 h); shift type, rotating schedule, and overtime hours were not collected separately. Self-rated personality type was collected using a single self-report item in which participants selected one of four categories (type A, type B, type C, or type D) according to their own perception. A validated personality inventory was not included because the original survey was designed to reduce respondent burden among busy ICU nurses and to collect broad contextual characteristics. Nevertheless, this single-item approach has limited psychometric validity. Therefore, findings related to personality type were interpreted as exploratory self-rated personality characteristics rather than clinically measured personality types and are acknowledged as a limitation. Grief State Scale for Nurses. The Chinese version translated and revised by Wang et al. (2023) was used. The instrument contains 17 items across four dimensions and two sub-scales: grief traits (emotional mood swings, 4 items; uncomfortable feelings toward deaths, 3 items) and grief state (feeling sadness toward patient deaths, 5 items; emotional exhaustion after caring, 5 items). Items are rated on a 5-point Likert scale from 1 (“not at all”) to 5 (“extremely so”), with higher scores indicating higher occupational grief. Cronbach's α and McDonald's ω for the total scale in this study were 0.932 and 0.933, respectively. Dimension-level Cronbach's α ranged from 0.852 to 0.937, and McDonald's ω ranged from 0.858 to 0.938.

#### Self-compassion scale-short form

The 12-item short form developed by Raes et al. (2011) was used. The scale covers six dimensions: self-kindness, self-judgment, common humanity, isolation, mindfulness, and over-identification. Items are rated on a 5-point Likert scale from 1 (“almost never”) to 5 (“almost always”). The negatively worded dimensions, including self-judgment, isolation, and over-identification, were reverse scored before calculating the total self-compassion score according to the scale instructions, so that higher total scores indicate higher levels of self-compassion. In the present data, Cronbach's α and McDonald's ω for the total scale were 0.868 and 0.837, respectively. Because each dimension of the short form contains only two items, dimension-level reliability coefficients should be interpreted cautiously; the common humanity dimension showed Cronbach's α = 0.638 and McDonald's ω = 0.640.

### Data collection

Data were collected through an online survey hosted on the WJX.cn platform. One research coordinator in each hospital was trained to distribute the electronic questionnaire link through departmental WeChat groups. The first page explained the study purpose, voluntary participation, procedures, anonymity protections, and informed consent. Each mobile phone number could be used only once. Two investigators independently reviewed returned questionnaires for completeness and logical consistency; questionnaires completed in an unrealistically short time or containing obvious logical errors were excluded. A total of 385 questionnaires were collected, of which 30 were excluded as invalid, leaving 355 valid questionnaires (valid response rate: 92.2%). No missing values were observed for the key demographic variables, occupational grief items, self-compassion items, or total/dimension scores in the final analytic sample, and complete-case analysis was therefore used. The online format may have introduced self-selection bias and common response bias; these issues were considered in interpretation.

### Statistical analysis

Data were analyzed using SPSS version 26.0 and R software. Categorical variables are presented as frequency and percentage, and continuous variables as mean ± standard deviation. Missing values were checked before analysis. Cronbach's alpha was calculated using SPSS. McDonald's omega and confirmatory factor analysis indices were obtained using R (psych and lavaan packages) as supplementary psychometric analyses. For the Self-Compassion Scale-Short Form, the negatively worded dimensions, including self-judgment, isolation, and over-identification, were reverse scored before calculating the total self-compassion score, so that higher total scores indicated higher levels of self-compassion. *Pearson* correlation analysis was used for summed total and dimension scores, which were treated as approximately continuous; *Spearman's* rank correlation was additionally performed as a sensitivity analysis because the items were measured using ordinal Likert responses. Independent-samples t tests and one-way analysis of variance were used for univariate comparisons of occupational grief across demographic and occupational subgroups. Effect sizes were reported for univariate comparisons, with Cohen's d for two-group comparisons and eta squared (η^2^) for comparisons involving more than two groups. Differences among dimension-level item mean scores were examined using Friedman tests with Bonferroni-adjusted pairwise Wilcoxon signed-rank tests. Hierarchical linear regression was performed according to theoretical relevance and empirical evidence. In Model 1, education level, working hours, self-rated health status, mindfulness/meditation-related activities, and self-rated personality type were entered. Self-compassion was entered in Model 2 as the psychological variable of primary theoretical interest. Dummy variables were created for education level, working hours, and personality type. Regression assumptions, including approximate normality, independence of residuals, homoscedasticity, and multicollinearity, were examined; variance inflation factors were within acceptable ranges. All tests were two tailed, and *p* < 0.05 was considered statistically significant.

## Results

### Common method variance

Harman's single-factor test was used to assess common method variance. Six factors with eigenvalues greater than 1 were extracted without rotation, and the first factor explained 30.67% of the variance (< 40%), indicating no serious common method bias in this study (Tang and Wen, 2020). Missing data, reliability, and factor-structure checks No missing values were found in the key variables used for analysis. The Grief State Scale for Nurses showed high internal consistency (Cronbach's α = 0.932; McDonald's ω = 0.933), and the dimension-level α*/*ω coefficients ranged from 0.852 to 0.937 and from 0.858 to 0.938, respectively. The four-factor CFA model of occupational grief showed CFI = 0.921, TLI = 0.904, RMSEA = 0.099, and SRMR = 0.073. For the Self-Compassion Scale-Short Form, after the negatively worded dimensions were reverse scored before total-score calculation, total-scale Cronbach's α = 0.868 and McDonald's ω = 0.837, whereas the six-factor CFA model showed CFI = 0.896, TLI = 0.824, RMSEA = 0.130, and SRMR = 0.084. These results support cautious interpretation of dimension-level self-compassion findings, especially the two-item common humanity dimension (Cronbach's α = 0.638; McDonald's ω = 0.640).

### Participant characteristics

Among the 355 ICU nurses, 53 (14.9%) were men and 302 (85.1%) were women. Participants were 20–49 years old, with a mean age of 31.39 ± 5.70 years. Most participants held a bachelor's degree (84.2%). Detailed demographic and occupational characteristics are shown in Table 1.

**Table 1:** Comparison of occupational grief among ICU nurses with different demographic characteristics (*n* = 355).

Variable	Category	Sample size (*n*)	Composition ratio (%)	Score (mean ±SD)	*t/F/r* value	*P* value	Effect size (*d*/η^2^)
Gender	Male	53	14.9	36.3 ± 15.28	0.761	0.447	*d* = 0.113
	Female	302	85.1	35.03 ± 10.33			
Age	≤ 25 years	68	19.2	35.93 ± 12.65	0.438	0.646	η^2^ = 0.002
	26–35 years	208	58.6	34.75 ± 10.58			
	≥36 years	79	22.3	35.85 ± 11.53			
Marital status	Unmarried	117	33	34.36 ± 11.47	0.978	0.377	η^2^ = 0.006
	Married	230	64.8	35.52 ± 11.08			
	Divorced	8	2.2	39.38 ± 10.05			
Working hours	< 8 h per shift	19	5.4	27.63 ± 6.12	7.357	< 0.001	η^2^ = 0.040
	8–10 h per shift	306	86.2	35.23 ± 10.61			
	>10 h per shift	30	8.5	40.00 ± 16.10			
Professional title	Nurse	74	20.8	35.49 ± 12.75	0.274	0.844	η^2^ = 0.002
	Nurse practitioner	118	33.2	35.73 ± 11.89			
	Charge nurse	153	43.1	34.63 ± 9.79			
	Associate chief nurse and above	10	2.8	36.40 ± 11.64			
Education level	Associate degree or below	43	12.1	37.02 ± 13.93	0.843	0.431	η^2^ = 0.005
	Bachelor's degree	299	84.2	35.06 ± 10.78			
	Master's degree and above	13	3.7	33.00 ± 10.52			
Received death education training	Yes	115	32.4	34.30 ± 11.19	−1.070	0.285	*d* = 0.121
	No	240	67.6	35.66 ± 11.19			
Frequency of mindfulness/meditation activities	Never	238	67.0	35.10 ± 11.95	2.755	0.042	η^2^ = 0.023
	Occasionally	85	23.9	36.58 ± 9.17			
	Sometimes	23	6.5	35.35 ± 9.68			
	Frequently	9	2.5	25.44 ± 6.72			
Self-rated health status	Poor	37	10.4	40.16 ± 14.33	9.052	< 0.001	η^2^ = 0.072
	Fair	192	54.1	36.77 ± 11.10			
	Good	112	31.5	31.48 ± 7.88			
	Excellent	14	3.9	30.93 ± 15.90			
Self-rated personality type	Type A	38	10.7	36.95 ± 8.93	13.827	< 0.001	η^2^ = 0.106
	Type B	235	66.2	32.87 ± 10.16			
	Type C	68	19.2	40.21 ± 10.38			
	Type D	14	3.9	45.86 ± 20.19			

Working hours represent self-reported usual total hours per shift. Shift type, rotating schedules, and overtime were not collected separately. Effect sizes were calculated from the final analytic dataset and are reported as Cohen's *d* for two-group comparisons and eta squared (η^2^) for comparisons involving more than two groups. For two-group comparisons, the absolute value of Cohen's *d* is shown.

### Occupational grief across demographic and occupational subgroups

Occupational grief differed significantly according to working hours, participation in mindfulness or meditation activities, self-rated health status, and self-rated personality type (all *p* < 0.05; Table 1). Effect sizes suggested that the differences by self-rated personality type and self-rated health status were larger than those for other demographic variables, whereas most non-significant comparisons showed very small effect sizes.

### Levels of occupational grief and self-compassion

The mean total occupational grief score was 35.22 ± 11.19, and the overall item mean score was 2.07 ± 0.66. In the absence of an established clinical cut-off, the item mean was descriptively interpreted as indicating a relatively mild level of occupational grief. Feeling sadness toward patient deaths had the highest item mean score (2.48 ± 0.76). Dimension-level item mean scores differed significantly across the four occupational grief dimensions (Friedman χ^2^ = 242.611, df = 3, *p* < 0.001). The mean total self-compassion score was 37.30 ± 4.03, and the overall item mean score was 3.11 ± 0.34. Self-kindness (3.70 ± 0.70) and mindfulness (3.66 ± 0.70) had the highest item mean scores among the self-compassion dimensions, and dimension-level item mean scores also differed significantly across the six self-compassion dimensions (Friedman χ^2^ = 656.508, df = 5, *P* < 0.001; Table 2). Occupational grief was negatively correlated with self-compassion in *Pearson* analysis (*r* = −0.323, *P* < 0.001), and this association remained significant in *Spearman* sensitivity analysis (ρ = −0.305, *P* < 0.001). Correlation coefficients for each dimension are presented in Table 3.

**Table 2:** Descriptive statistics for occupational grief and self-compassion among ICU nurses (*n* = 355).

Variable	No. of items	Possible total score range	Observed total score (mean ±SD)	Item mean score (mean ±SD)
Occupational grief total score	17	17–85	35.22 ± 11.19	2.07 ± 0.66
Emotional mood swings	4	4–20	8.10 ± 3.74	2.02 ± 0.93
Uncomfortable feelings toward deaths	3	3–15	5.89 ± 2.73	1.96 ± 0.91
Feeling sadness toward patient deaths	5	5–25	12.43 ± 3.79	2.48 ± 0.76
Emotional exhaustion	5	5–25	8.81 ± 3.76	1.76 ± 0.75
Self-compassion total score	12	12–60	37.30 ± 4.03	3.11 ± 0.34
Self-kindness	2	2–10	7.40 ± 1.40	3.70 ± 0.70
Mindfulness	2	2–10	7.34 ± 1.41	3.66 ± 0.70
Common humanity	2	2–10	6.41 ± 1.59	3.20 ± 0.79
Isolation	2	2–10	5.41 ± 1.60	2.70 ± 0.80
Over-identification	2	2–10	4.92 ± 1.41	2.46 ± 0.70
Self-judgment	2	2–10	5.82 ± 1.66	2.90 ± 0.83

Item mean score was calculated by dividing the total score by the number of items in the corresponding scale or dimension. Friedman tests were used to compare dimension-level item mean scores. The occupational grief dimensions differed significantly across dimensions (Friedman χ^2^ = 242.611, df = 3, *P* < 0.001), and the self-compassion dimensions also differed significantly across dimensions (Friedman χ^2^ = 656.508, df = 5, *P* < 0.001). Bonferroni-adjusted *post-hoc* paired Wilcoxon signed-rank tests were used where appropriate.

**Table 3:** Correlation analysis of occupational grief and self-compassion among ICU nurses (*n* = 355).

Variable	Emotional mood swings	Uncomfortable feelings toward deaths	Feeling sadness toward patient deaths	Emotional exhaustion	Occupational grief total score
Self-kindness	−0.240^**^	−0.059	0.013	−0.115^*^	−0.129^*^
Mindfulness	−0.195^**^	−0.044	0.029	−0.072	−0.09
Common humanity	0.023	0.106^*^	0.107^*^	0.132^*^	0.114^*^
Isolation	−0.230^**^	−0.199^**^	−0.118^*^	−0.197^**^	−0.232^**^
Over-identification	−0.160^**^	−0.209^**^	−0.235^**^	−0.185^**^	−0.246^**^
Self-judgment	−0.174^**^	−0.278^**^	−0.173^**^	−0.263^**^	−0.273^**^
Total self-compassion total score	−0.363^**^	−0.262^**^	−0.144^**^	−0.265^**^	−0.323^**^

Values in the table are Pearson correlation coefficients based on summed dimension and total scores. Spearman sensitivity analysis showed that occupational grief remained negatively associated with self-compassion (ρ = −0.305, *P* < 0.001), while common humanity was not significantly associated with total occupational grief (ρ = 0.099, *P* = 0.062). ^**^P < 0.01; ^*^P < 0.05.

### Hierarchical regression analysis

The coding of the independent variables used in the regression analysis is shown in Table 4. In Model 1, theoretically relevant demographic, occupational, health-related, and personality variables, including education level, self-rated health status, working-hour dummy variables, mindfulness or meditation activities, and self-rated personality type, explained 16.1% of the variance in occupational grief (adjusted *R*^2^ = 0.139). After self-compassion was added in Model 2, the total explained variance increased to 20.2% (adjusted *R*^2^ = 0.179), with self-compassion contributing an additional 4.1% (*F* change *P* < 0.001; Table 5). Poorer self-rated health, self-rated type C personality, self-rated type D personality, and lower self-compassion were independently associated with higher occupational grief. Regarding working-hour categories, nurses working fewer than 8 h per duty day reported lower occupational grief than those working 8–10 h, whereas the >10-h category did not reach statistical significance in the adjusted model. Education level was not significantly associated with occupational grief in the adjusted model. Multicollinearity was not evident (maximum VIF = 1.231).

**Table 4:** Coding of independent variables for regression analysis.

Variable	Variable type	Coding/assigned value
Education level	Dummy-coded categorical variable	Bachelor's degree was used as the reference category. Junior college or below: 1 = junior college or below, 0 = other; master's degree or above: 1 = master's degree or above, 0 = other.
Working-hour category	Dummy-coded categorical variable	Working 8–10 h per shift was used as the reference category. < 8 h: 1 = < 8 h, 0 = other; >10 h: 1 = >10 h, 0 = other. Working hours referred to self-reported usual total working hours per duty day.
Self-rated health status	Ordinal covariate	Poor = 1; fair = 2; good = 3; excellent = 4; higher scores indicate better health.
Frequency of mindfulness/ meditation activities	Ordinal covariate	Never = 1; occasionally = 2; sometimes = 3; frequently = 4; higher scores indicate greater frequency.
Self-rated personality type	Dummy-coded categorical variable	Self-rated Type B was used as the reference category. Self-rated Type A: 1 = Type A, 0 = other; self-rated Type C: 1 = Type C, 0 = other; self-rated Type D: 1 = Type D, 0 = other.
Total self-compassion score	Continuous variable	The total score of the self-compassion scale-short Form was entered as a continuous variable. Negatively worded dimensions, including self-judgment, isolation, and over-identification, were reverse scored before calculating the total score; higher scores indicate higher self-compassion.

The dependent variable was the total occupational grief score. Bachelor's degree, working 8–10 h per shift, and self-rated Type B were used as the reference categories.

**Table 5:** Hierarchical linear regression analysis of occupational grief among ICU nurses (*n* = 355).

Variable	Model 1 *b* (95% CI)	Model 1 *p*	Model 2 *b* (95% CI)	Model 2 *p*
Self-rated health status	−2.389 (−4.060, −0.718)	0.005	−2.051 (−3.691, −0.411)	0.014
Mindfulness/meditation activities	−0.505 (−2.003, 0.994)	0.508	−0.175 (−1.646, 1.297)	0.816
Education: college or below vs. bachelor's degree	2.377 (−0.985, 5.740)	0.165	2.058 (−1.230, 5.345)	0.219
Education: master's degree or above vs. bachelor's degree	−0.742 (−6.702, 5.218)	0.807	0.592 (−5.261, 6.446)	0.842
Working hours: < 8 vs. 8–10 h	−5.709 (−10.622,−0.796)	0.023	−5.843 (−10.641, −1.045)	0.017
Working hours: >10 vs. 8–10 h	3.656 (−0.335, 7.647)	0.072	3.305 (−0.596, 7.206)	0.097
Personality type: Type A vs. Type B	3.428 (−0.225, 7.082)	0.066	3.073 (−0.498, 6.645)	0.091
Personality type: Type C vs. Type B	5.505 (2.560, 8.450)	< 0.001	3.780 (0.794, 6.767)	0.013
Personality type: Type D vs. Type B	11.286 (5.541, 17.031)	< 0.001	8.896 (3.176, 14.616)	0.002
Self-compassion total score	/	/	−0.615 (−0.902, −0.328)	< 0.001
*F*	7.331^***^		8.695^***^	
*R* ^2^	0.161		0.202	
Delta *R*^2^	0.161		0.041	

Model 1 included education level, working hours, self-rated health status, mindfulness/meditation-related activities, and self-rated personality type. Model 2 added total self-compassion score. Bachelor's degree, 8–10 working hours per duty day, and Type B personality were reference categories for dummy variables. Maximum VIF = 1.231. ^***^P < 0.001.

## Discussion

### Occupational grief and self-compassion among ICU nurses

This study found that occupational grief among ICU nurses was relatively mild based on the overall item mean score, while self-compassion was at a moderate level. This interpretation should be made cautiously because the Grief State Scale for Nurses has no established clinical cut-off values. Nevertheless, the finding is consistent with previous Chinese studies reporting measurable occupational grief among nurses (Xiong et al., 2023; Feng et al., 2023). Feeling sadness toward patient deaths had the numerically highest dimension score. Because no inferential comparison across dimensions was performed, this descriptive pattern should not be interpreted as demonstrating a statistically higher level than the other dimensions. Repeated exposure to critically ill patients and patient death may be one possible explanation, but these exposures were not directly quantified in the present study.

Occupational grief was associated with working-hour category, poorer self-rated health, and self-rated type C or type D personality. However, the adjusted model did not support a simple linear interpretation that longer working hours were independently associated with higher occupational grief. Compared with nurses working 8–10 h per duty day, those working fewer than 8 h reported lower occupational grief, whereas the association for working more than 10 h did not reach statistical significance. Shorter duty periods may allow more recovery time, although the present study measured only usual total hours per duty day rather than detailed shift schedules, night shifts, or overtime (Dall'Ora et al., 2023; Li et al., 2022). Nurses with poorer self-rated perceived health may have lower perceived recovery capacity, which could make grief-related distress more difficult to manage. Because personality type was assessed by a self-report item rather than a validated personality inventory, these findings should be interpreted cautiously. Nurses who self-identified as type C or type D showed higher occupational grief, but the present design cannot confirm underlying traits such as emotional suppression or negative affect Matsuishi et al. (2022). The non-significant association between self-rated type A personality and occupational grief may be related to the use of a single self-identification item, the relatively limited ability of broad self-rated categories to capture stress reactivity, and the possibility that the high-pressure ICU environment reduces between-group differences in grief responses.

### Association between self-compassion and occupational grief

Higher self-compassion was associated with lower occupational grief, and self-compassion remained independently associated with occupational grief in hierarchical regression. This finding directly addresses the study question regarding the relationship between self-compassion and occupational grief. The association is consistent with the theoretical view that self-compassion may help individuals respond to suffering with self-kindness, balanced awareness, and reduced over-identification (Neff, 2023). In ICU practice, it is plausible that these capacities relate to less self-blame or rumination after patient death, but this mechanism was not measured in the present study. Previous meta-analyses and reviews have linked self-compassion with lower psychological distress and better psychological outcomes (Suh and Jeong, 2021; Ferrari et al., 2019; Hughes et al., 2021). Studies in ICU and broader nursing populations also suggest that self-compassion is related to professional quality of life and psychological wellbeing (Moreno-Jiménez et al., 2022; Wu et al., 2024; Bissett, 2022).

The association observed in this study should be interpreted as correlational rather than causal. The present cross-sectional data cannot demonstrate that self-compassion reduces occupational grief or that self-compassion training, reflective practice, or grief-support programs improve outcomes. The final regression model explained 20.2% of the variance in occupational grief, indicating that approximately four fifths of the variance remained unexplained. Important unmeasured factors, such as detailed shift schedules, ICU workload intensity, patient mortality exposure, staffing ratio, organizational support, ethical climate, moral distress, leadership support, team climate, and grief-support resources, may also contribute to occupational grief. Therefore, self-compassion may be considered a potentially relevant psychological correlate (Yang and Chen, 2021), but future longitudinal and intervention studies are needed to test causal pathways and practical intervention effects.

From a practical perspective, organizations may cultivate self-compassion and grief coping by providing structured debriefing after patient death, reflective practice sessions, peer-support groups, mindfulness- or compassion-based education, grief-support workshops, and accessible psychological support. Such strategies should not place responsibility solely on individual nurses; they should be implemented together with organizational measures that address workload, staffing, team communication, ethical support, and bereavement-support culture (Jit Singh et al., 2023). Because these interventions were not tested in the present study, they should be regarded as potential directions for future research rather than confirmed implications of the current cross-sectional data.

### Interpretation of specific correlation findings and cultural considerations

Table 3 showed small positive *Pearson* correlations between common humanity and some occupational grief dimensions. Item coding was rechecked against the scale instructions: common humanity items are positively keyed and were not reverse scored. However, the common humanity dimension contains only two items and showed moderate internal consistency (Cronbach's α = 0.638; McDonald's ω = 0.640). In the Spearman sensitivity analysis, common humanity was not significantly associated with total occupational grief (ρ = 0.099, *P* = 0.062), although small positive associations with feeling sadness toward patient deaths and emotional exhaustion after caring approached statistical significance. Therefore, these findings should not be interpreted as evidence that common humanity increases occupational grief. They may instead reflect measurement limitations of a short two-item subscale, greater emotional awareness of shared suffering, empathic engagement, or item wording effects; these hypotheses require further psychometric and qualitative investigation.

The present study did not measure cultural norms, organizational climate, or workplace grief-support practices. Cultural and organizational explanations for grief expression among Chinese ICU nurses therefore should not be treated as findings of this study. It is possible that professional expectations or local norms influence whether grief is discussed openly, but this remains a hypothesis for future qualitative and multicenter research.

### Limitations

This study has several limitations. First, the cross-sectional design precludes causal inference and prevents conclusions about the direction of the association between self-compassion and occupational grief. Second, all data were self-reported and collected using the same online questionnaire, which may introduce recall bias, social desirability bias, and common method bias, although Harman's single-factor test did not indicate serious common method variance. Third, online data collection may have introduced self-selection bias and possible survivor bias, because nurses who were absent, on leave, experiencing greater distress, or unwilling to participate may have differed from respondents. Fourth, hospitals were not randomly selected and were limited to one province, which restricts generalizability beyond tertiary hospitals in Shandong Province. Fifth, ICU-level characteristics were not adjusted for, including staffing ratio, patient mortality exposure, unit workload, organizational support, ethical climate, moral distress, and bereavement-support resources. Sixth, personality type was assessed using a single self-rated item rather than a validated personality inventory; thus, personality-related findings should be interpreted as exploratory associations rather than evidence of measured personality traits. Seventh, although education level was included in the revised regression model, the model explained only part of the variance in occupational grief. Eighth, the Self-Compassion Scale-Short Form contains short two-item dimensions and showed less optimal CFA fit in this sample; dimension-level findings, particularly common humanity, should therefore be interpreted cautiously. Finally, future studies should use validated personality measures, more detailed shift-work and ICU-level indicators, longitudinal designs, and robust psychometric testing.

## Conclusion

Occupational grief among ICU nurses in tertiary hospitals was relatively mild based on item mean scores, while self-compassion showed a moderate level; these descriptions are not clinical classifications because validated cut-off values are unavailable. Poorer self-rated health, longer working hours, self-rated type C personality, self-rated type D personality, and lower self-compassion were independently associated with higher occupational grief after education level was additionally considered. For working-hour categories, working fewer than 8 h per duty day was associated with lower occupational grief compared with working 8–10 h, whereas the >10-h category was not statistically significant in the adjusted model. Because personality type was self-rated and the regression model explained only part of the variance, these findings should be interpreted cautiously. Future longitudinal and intervention studies are needed to examine whether self-compassion-focused and organizational grief-support strategies are effective in alleviating occupational grief among ICU nurses.

## Data Availability

The original contributions presented in the study are included in the article/supplementary material, further inquiries can be directed to the corresponding author.
